# RNA-Seq-Based Transcriptomics and GC–MS Quantitative Analysis Reveal Antifungal Mechanisms of Essential Oil of *Clausena lansium* (Lour.) Skeels Seeds against *Candida albicans*

**DOI:** 10.3390/molecules28248052

**Published:** 2023-12-12

**Authors:** Yinzheng Ma, Jinlei Sui, Yan Wang, Wanying Sun, Guohui Yi, Jinyan Wu, Shi Qiu, Lili Wang, Aihua Zhang, Xiaowen He

**Affiliations:** 1 Public Research Center, Hainan Medical University, Haikou 571199, China; hbykdxmyz@163.com (Y.M.); sui3com@126.com (J.S.); 15776588894@163.com (Y.W.); wanying998@126.com (W.S.); guohuiyi6@hainmc.edu.cn (G.Y.); jinyanzi0720@126.com (J.W.); qiushihnyx@163.com (S.Q.); wanglili0310211@163.com (L.W.); 2 School of Pharmacy, Hainan Medical University, Haikou 571199, China; 3 Key Laboratory of Emergency and Trauma of Ministry of Education, Research Unit of Island, Emergency Medicine of Chinese Academy of Medical Sciences, Hainan Medical University, Haikou 571199, China

**Keywords:** *Clausena lansium* (Lour.) Skeels seeds, essential oil, *Candida albicans*, drug resistance, gas chromatography–mass spectrometry, transcriptomics analysis

## Abstract

Infections caused by *Candida albicans* (*C. albicans*) and increasing resistance to commonly used drugs lead to a variety of mucosal diseases and systemic infectious diseases. We previously confirmed that the essential oil of *Clausena lansium* (Lour.) Skeels seeds (CSEO) had antifungal activity against *C. albicans*, but the detailed mechanism between the chemical components and antifungal activity is unclear. In this study, a quantitative analysis of five volatile components of CSEO, including sabinene, *α*-phellandrene, *β*-phellandrene, 4-terpineol, and *β*-caryophyllene, was carried out using the gas chromatography–mass spectrometry (GC–MS) method. Both the broth dilution and kinetic growth methods proved that the antifungal activity of CSEO against fluconazole-resistant *C. albicans* was better than that of its main components (sabinene and 4-terpineol). To further investigate the inhibitory mechanism, the transcriptional responses of *C. albicans* to CSEO, sabinene, and 4-terpineol treatment were determined based on RNA-seq. The Venn diagram and clustering analysis pattern of differential expression genes showed the mechanism of CSEO and 4-terpineol’s anti-*C. albicans* activity might be similar from the perspective of the genes. Functional enrichment analysis suggested that CSEO regulated adherence-, hyphae-, and biofilm-formation-related genes, which may be CSEO’s active mechanism of inhibiting the growth of fluconazole-resistant *C. albicans*. Overall, we preliminarily revealed the molecular mechanism between the chemical components and the antifungal activity of CSEO against *C. albicans.* This study provides new insights to overcome the azole resistance of *C. albicans* and promote the development and application of *C. lansium* (Lour.) Skeels seeds.

## 1. Introduction

*Candida* species are the main opportunistic pathogenic fungi of humans [1]. Invasive candidiasis caused by *Candida albicans* (*C. albicans*) is a major problem in the healthcare field that leads to high mortality rates (40–60%) [2]. The risk of disseminated candidiasis has increased due to the wide application of broad-spectrum antibiotics, glucocorticoids, and immunosuppressants, especially because of their increased use in AIDS and cancer patients. It was reported that *C. albicans* was the primary pathogen of invasive *Candida* infection (74.29%) in patients with perforated peptic ulcers who received laparotomies or laparoscopic surgery in Taiwan, China [3]. In addition, the mortality rate of sepsis (approximately 40%) caused by *C. albicans* is higher than that of any other sepsis caused by fungi or bacteria in the USA [4].

The azoles represented by fluconazole, with a broad antimicrobial spectrum, long half-life, high bioavailability, and few adverse reactions, have become the most common antifungal agents for treating *C. albicans* infection. However, drug resistance has significantly increased due to the general and long-term use of azoles in clinics [5]. Therefore, to address the issue of antifungal drug resistance, the potential mechanisms of antifungal drug resistance and two or more antifungal drugs used in combination have become hot topics [6,7,8]. Furthermore, another approach is searching for new active antifungal components from natural or traditional medicines to overcome antifungal drug resistance [9,10,11]. Essential oils (EOs) have attracted attention due to their unique physicochemical properties and biological activities. For example, the EO of *Hyssopus officinalis* L. from Bulgaria increased yeast membrane permeability and disrupted normal membrane transport by affecting membrane ATPase. It is active against both fluconazole-sensitive and fluconazole-resistant clinical *Candida* spp. [12]. The EO of *Thymus vulgaris* and its major active component, thymol, have strong antifungal activity against *Candida* spp. by inhibiting and eradicating biofilms [13].

*Clausena lansium* (Lour.) Skeels, belonging to the Rutaceae family, is indigenous to and commonly cultivated in tropical and subtropical regions of China such as Hainan, Fujian, and Guangxi [14]. In traditional Chinese medicine, the fruit of *C. lansium* (Lour.) Skeels has the effects of eliminating digestive stagnation, relieving gastrointestinal gas, dissipating heat, and relieving pain [15,16]. People usually eat *C. lansium* (Lour.) Skeels fruit fresh or make it into juice, preserves, or jam. However, *C. lansium* (Lour.) Skeels seeds, as a by-product of pulp processing, are mostly discarded. This not only leads to resource waste but also causes environmental pollution. Therefore, studies on the components and biological activities of *C. lansium* seeds are increasing [17,18,19]. There are many components, including alkaloids, volatile components, and coumarins, in *C. lansium* (Lour.) Skeels seeds. Their extensive biological activities have attracted widespread attention, including their anticancer, antidiabetic, anti-inflammatory, insecticidal, and antioxidant effects [20,21,22].

In our previous study, the comparative percentages of components of essential oil of *Clausena lansium* (Lour.) Skeels seeds (CSEO) were determined using the gas chromatography–mass spectrometry (GC-MS) method [21]. Each component was identified by comparing its relative retention index and the NIST library database. The relative contents of the components were computed using the peak areas without applying correction factors. The disc diffusion method showed the antifungal activity of CSEO against *Candida* spp. (*C. albicans*, *C. krusei*, *C. tropicalis*, *C. parapsilosis,* and *C. glabrata*) with inhibition zone diameters of 9.4–23.4 mm against both fluconazole-sensitive and fluconazole-resistant strains. Comprehensive analysis indicated that the chemical components might be positively related to anti-*Candida* activity, but this has not been verified.

To investigate the relationship between the chemical components and anti-*C. albicans* activity of CSEO, quantitative analysis of five volatile components of CSEO, including sabinene, *α*-phellandrene, *β*-phellandrene, 4-terpineol, and *β*-caryophyllene, was performed using a standard curve method and GC–MS. The transcriptional responses of *C. albicans* to CSEO, sabinene, and 4-terpineol treatment were determined based on RNA-seq to reveal the inhibitory mechanism of CSEO.

## 2. Results and Discussion

### 2.1. CSEO Extraction and Analysis

**Figure 1:**
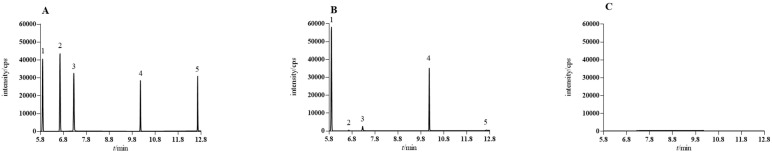
Chromatograms of standard solution (**A**), sample solution (**B**), and blank solution (**C**) using GC–MS. (1, sabinene; 2, *α*-phellandrene; 3, *β*-phellandrene; 4, 4-terpineol; 5, *β*-caryophyllene).

The extracted CSEO was almost transparent in color. The overall yield was 0.68 ± 0.05 (*v*/*w*). Firstly, a standard stock solution containing sabinene, *α*-phellandrene, *β*-phellandrene, 4-terpineol, and *β*-caryophyllene was prepared in *n*-hexane. The retention time of each component was obtained by injecting the standard solution via GC–MS. In the specificity test, the peaks of sabinene, *α*-phellandrene, *β*-phellandrene, 4-terpineol, and *β*-caryophyllene in CSEO were well isolated, and there was no interference component (Figure 1). Eight standard stock solutions were prepared for the establishment of standard curves. Good linearity was obtained for each component using the peak area of each component as the vertical axis and the corresponding concentration as the horizontal axis. The limit of quantification (LOQ) and limit of detection (LOD) were calculated by using signal-to-noise ratios (S/N) of 10 and 3, respectively. Table 1 lists the retention times, linear ranges, slopes, intercepts, correlation coefficients (*r*), LOQs, and LODs.

To evaluate the repeatability and stability of the proposed method, CSEO solutions in six independent series were analyzed on the same day. The relative standard deviation values (RSDs) of the peak areas for precision were 0.28%, 0.31%, 0.36%, 0.28%, and 0.32% for sabinene, *α*-phellandrene, *β*-phellandrene, 4-terpineol, and *β*-caryophyllene, respectively. The CSEO solutions were stable for 24 h at 4 °C with RSDs of 0.26%, 0.28%, 0.36%, 0.33%, and 0.42% for the five components. The accuracy of the developed method was determined based on the percentages of recovery. The recovery analysis results were 100.21%, 99.55%, 100.33%, 99.74%, and 100.55% for sabinene, *α*-phellandrene, *β*-phellandrene, 4-terpineol, and *β*-caryophyllene, respectively, showing excellent recoveries, with RSDs ranging from 1.30% to 1.49%. Thus, the developed method is simple, accurate, and suitable for determining these five components of CSEO. Finally, the contents of sabinene, *α*-phellandrene, *β*-phellandrene, 4-terpineol, and *β*-caryophyllene were tested, obtaining values of 177.31 ± 5.23 mg·mL^−1^, 2.69 ± 0.18 mg·mL^−1^, 11.70 ± 0.45 mg·mL^−1^, 83.30 ± 2.36 mg·mL^−1^, and 1.51 ± 0.14 mg·mL^−1^, respectively. Among these five components, sabinene and 4-terpineol were the main components of CSEO.

In our previous study, we found significant differences in the types and relative contents of volatile components derived from *C. lansium* using different extraction methods. A total of 33 volatile components were detected in *C. lansium* seeds from six different regions in Hainan, China, using the ionic-liquid-based headspace gas chromatography–mass spectrometry (HS–GC–MS) method [22]. The main volatile components were sabinene (17.76–43.81%), *β*-bisabolene (5.83–22.25%), and caryophyllene (6.75–16.62%). Forty-six volatile components were identified in thirteen EOs extracted via hydrodistillation using GC–MS [21]. The volatile components were rich in sabinene (27.70–54.28%) and accompanied by *β*-phellandrene (13.39–25.91%) and 4-terpineol (6.33–11.83%). The components and contents greatly differed using different extraction methods. There were also differences in the components and contents using the same hydrodistillation method. For example, 26 volatile components were identified in the EO of *C. lansium* seeds from Changjiang County, Hainan, China [20]. The main components were phellandrene (54.8%), limonene (23.6%), and 4-terpineol (7.5%). The differences may be related to the origin, harvest time, drying method, and other factors. However, one of the most important reasons may be that all the above studies identified the volatile components in *C. lansium* by comparing the GC–MS library database and the relative retention indices. These previous obtained contents of the components were calculated using the normalization method of the peak areas without applying correction factors. The contents were relative, not absolute, which led to the significant differences.

In this study, a standard curve method using GC–MS was developed for the quantitative analysis of five volatile components of CSEO, including sabinene, *α*-phellandrene, *β*-phellandrene, 4-terpineol, and *β*-caryophyllene. The method was simple, rapid, sensitive, and specific, with high accuracy. Finally, the contents of sabinene, *α*-phellandrene, *β*-phellandrene, 4-terpineol, and *β*-caryophyllene were tested. The content results of these five components differed from their relative contents, which is one of the reasons for establishing the quantitative method.

### 2.2. Antifungal Effects of Volatile Components

The MICs of sabinene, 4-terpineol, and CSEO were 1.00, 2.00, and 1.00 mg·mL^−1^ against *C. albicans* SC5314 and 0.50, 1.00, and 0.25 mg·mL^−1^ against *C. albicans* 27, respectively. The MFCs were a little higher than the corresponding MICs. The MFCs of sabinene to *C. albicans* SC5314 and *C. albicans* 27 were 2.00 and 1.00 mg·mL^−1^, respectively. The MFCs of 4-terpineol to *C. albicans* SC5314 and *C. albicans* 27 were 4.00 and 2.00 mg·mL^−1^, respectively. The MFCs of CSEO to *C. albicans* SC5314 and *C. albicans* 27 were 2.00 and 0.50 mg·mL^−1^, respectively. Notably, the MICs and MFCs of sabinene, 4-terpineol, and CSEO against the fluconazole-resistant strain *C. albicans* 27 were all lower than those against *C. albicans* SC5314.

**Figure 2:**
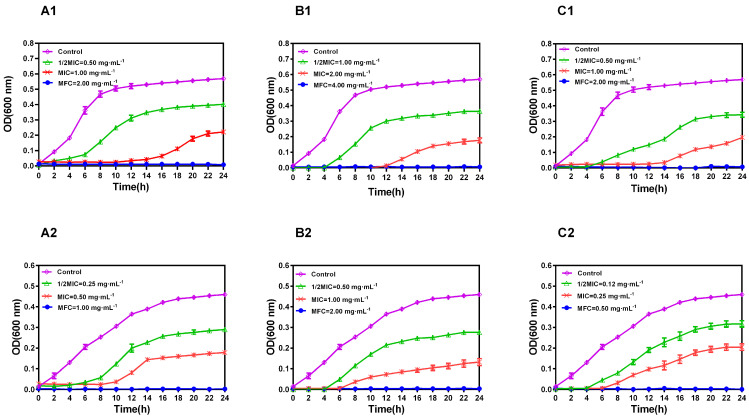
Growth kinetics of *C. albicans* SC5314 (**A1**–**C1**) and *C. albicans* 27 (**A2**–**C2**) in absence (C◇) and presence of ½ MIC (△), MIC (x), and MFC (●) (mg·mL^−1^) of sabinene (**A1**,**A2**), 4-terpineol (**B1**,**B2**), and CSEO (**C1**,**C2**), respectively. Data represent the mean values of three measures of the optical density at 600 nm.

The growth kinetics of *C. albicans* SC5314 and *C. albicans* 27 in the presence of sabinene, 4-terpineol, and CSEO (Figure 2) showed that these chemicals and CSEO have different degrees of influence on the growth trend of *C. albicans* at concentrations of 0.25–4.00 mg·mL^−1^. As shown in Figure 2, sabinene at a concentration of 2.00 mg·mL^−1^, 4-terpineol at 4.00 mg·mL^−1^, and CSEO at 2.00 mg·mL^−1^ inhibit the growth of *C. albicans* SC5314. Moreover, the growth of *C. albicans* 27 was inhibited with concentrations of 1.00 mg·mL^−1^ (sabinene), 2.00 mg·mL^−1^ (4-terpineol), and 0.50 mg·mL^−1^ (CSEO). The growth curves show that CSEO has a better inhibitory effect on fluconazole-resistant *C. albicans* than the two pure chemicals.

There are few studies on the antifungal activity of *C. lansium* seed extracts against *Candida* strains. Yan et al. reported that two amides, lansiumamide B and lansiumamide C, isolated from *C. lansium* seeds displayed antifungal activity against *Sclerotinia sclerotiorum* [17]. The curative efficacy (75.17%) of lansiumamide B was better than that of the positive drug carbendazim (56.57%) against *S. sclerotiorum* infection, proving that it can cause cell rupture and abnormal hyphae in *S. sclerotiorum*. In our previous study, the antifungal activities of CSEO against five *Candida* strains (*C. albicans*, *C. tropicalis*, *C. krusei*, *C. glabrata*, and *C. parapsilosis*) were preliminarily investigated [21]. The inhibition zone diameters of CSEO using the disc diffusion method were in the range of 9.4–23.4 mm. Significantly, the CSEO were active against fluconazole-resistant strains. Therefore, to investigate the relationship between the chemical components and antifungal activity, the main volatile components (sabinene and 4-terpineol) of CSEO were obtained via GC–MS, and the antifungal activities of CSEO, sabinene, and 4-terpineol against *C. albicans* were studied using the broth dilution method and the growth curve method. The results of both methods showed that the antifungal activity of CSEO against fluconazole-resistant *Candida albicans* was better than that of its main components, sabinene and 4-terpineol.

### 2.3. Analysis of DEGs

To further evaluate the inhibitory mechanisms of CSEO against *C. albicans*, we performed a transcriptome analysis using RNA-seq technology. The transcriptome databases were obtained to investigate the antifungal mechanism of CSEO, sabinene, and 4-terpineol against *C. albicans*. We generated a total of 1,187,604,202 raw reads and 1,187,519,206 clean reads. The average error rate of all presented data was less than 0.03%. Table S1 provides an overview of the transcriptome assembly statistics. The gene expression levels under different experimental treatments were compared in terms of the FKPM values. The FKPM results show there was no difference in the expression level in each sample.

**Figure 3:**
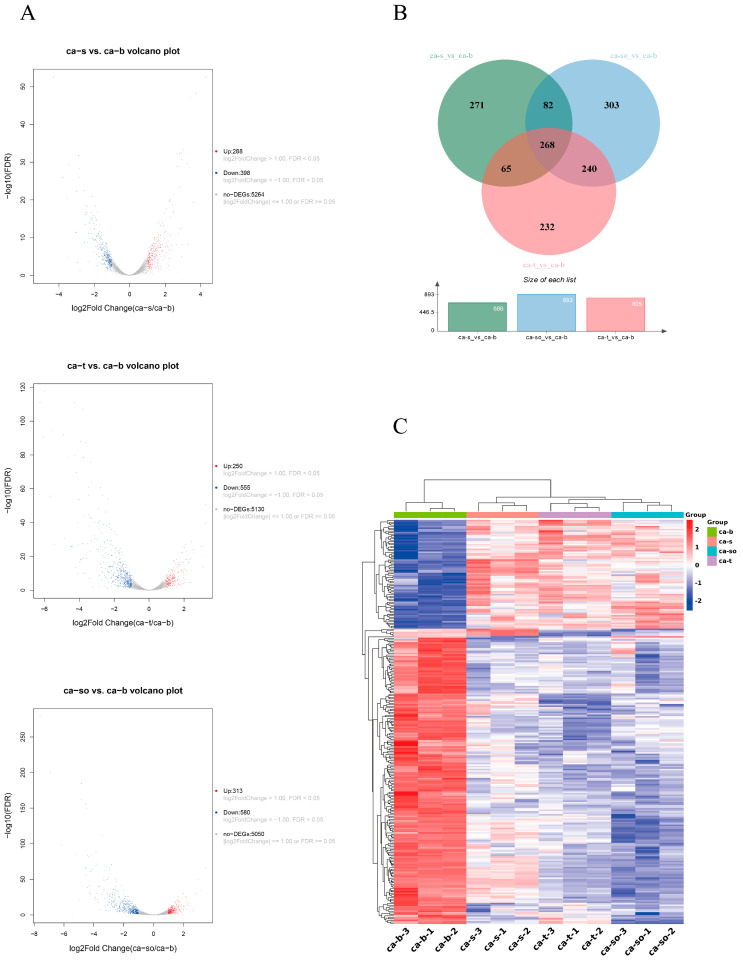
(**A**) Volcano plots of DEGs for ca-s vs. ca-b, ca-t vs. ca-b, and ca-so vs. ca-b. Down-regulated genes are represented using blue dots, up-regulated genes are represented using red dots, and genes without differential expression are represented using gray dots. (**B**) Venn diagram of DEGs for ca-s vs. ca-b, ca-t vs. ca-b, and ca-so vs. ca-b. (**C**) Clustering heat map of expression levels for all identical DEGs of ca-b, ca-s, ca-t, and ca-so. “ca-b-1, ca-b-2, ca-b-3” represent three biological replicates of ca-b. Others are the same as ca-b. The transition from blue to red bands indicates an increase in gene expression levels.

*C. albicans* SC5314 cells exposed to the MICs of sabinene (ca-s), 4-terpineol (ca-t), and CSEO (ca-so) for 1 h were collected for the RNA-seq, respectively. At the same time, untreated *C. albicans* SC5314 cells (ca-b) were used as the control. To evaluate the transcription, the three experimental groups (ca-s, ca-t, and ca-so) and the control group (ca-b) were compared. The volcano plots illustrating the DEG distribution of the three experimental groups and the control group are shown in Figure 3A. A total of 686 and 805 significant DEGs were identified in ca-s vs. ca-b and ca-t vs. ca-b, respectively. There were 288 up-regulated genes and 398 down-regulated genes in ca-s vs. ca-b, and 250 up-regulated genes, and 555 down-regulated genes in ca-t vs. ca-b. A total of 893 DEGs was identified in ca-so vs. ca-b. Among them, 580 were down-regulated genes and 313 were up-regulated genes.

A Venn diagram was created to compare the logical relationship between the DEGs in the three experimental groups, as shown in Figure 3B. A total of 268 identical DEGs among the three groups indicated that the anti-*C. albicans* activity of CSEO was related to sabinene and 4-terpineol. In addition, CSEO shared 240 identical DEGs with 4-terpineol and 82 identical DEGs with sabinene, which shows that 4-terpineol might play a more prominent role than sabinene in the anti-*C. albicans* activity of CSEO. All 268 identical DEGs in the three experimental groups were selected to determine the differential gene expression pattern among the three groups via clustering analysis (Figure 3C). The regions of different colors represent different clustering information. If the gene expression patterns are similar, there are similar or identical biological processes in the same group. As seen in Figure 3C, three biological replicates in each group had similar gene expression patterns, but all three groups were significantly different from the control (ca-b). CSEO and 4-terpineol were grouped, exhibiting the highest correlation. These results show that the mechanism of the anti-*C. albicans* activity of CSEO and 4-terpineol might be similar from the perspective of genes.

### 2.4. GO and KEGG Enrichment Analysis

**Figure 4:**
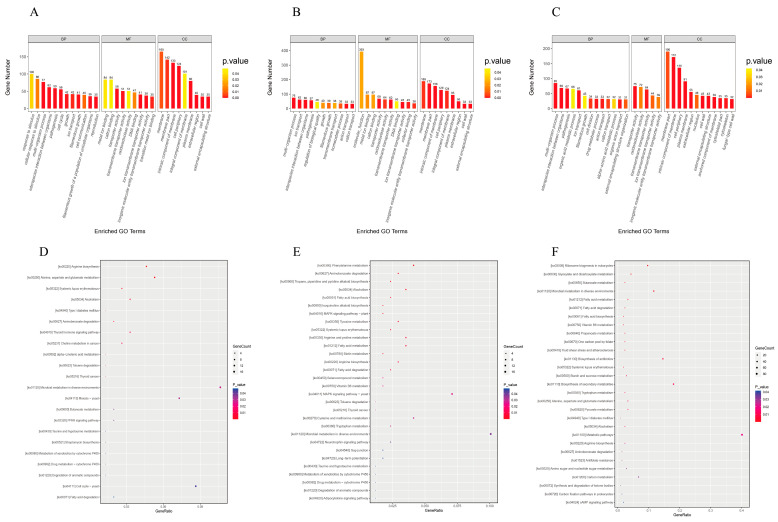
GO and KEGG enrichment analysis of DEGs between the three experimental groups and control. The top 30 significantly enriched GO terms for *C. albicans* exposed to sabinene (**A**), 4-terpineol (**B**), and CSEO (**C**) and scatter plots of all significantly enriched KEGG pathways for *C. albicans* treated with sabinene (**D**), 4-terpineol (**E**), and CSEO (**F**).

GO enrichment analysis was performed to investigate the DEG functions, including biological processes (BP), cellular components (CC), and molecular function (MF). The top 30 significantly enriched GO terms between the three experimental groups and the control group were selected and are displayed in Figure 4A–C. The top 30 significantly enriched GO terms for *C. albicans* exposed to sabinene are shown in Figure 4A. There were 12, 9, and 9 subcategories in BP, MF, and CC, respectively. Figure 4B shows the significantly enriched GO terms for *C. albicans* exposed to 4-terpineol, including 11, 10, and 9 subcategories in BP, CC, and MF. The top 30 significantly enriched GO terms between CSEO and the control are shown in Figure 4C, including 13, 5, and 12 subcategories in BP, MF, and CC. The GO terms for *C. albicans* treated with sabinene have five, seven, and five identical subcategories with CSEO in BP, MF, and CC, respectively. The GO terms for *C. albicans* treated with 4-terpineol have seven, five, and eight identical subcategories with CSEO in BP, MF, and CC, respectively. This indicates that the anti-*C. albicans* activity of CSEO has close relationships with the two chemicals, and the relationship between CSEO and 4-terpineol is closer than with sabinene. We also found that there were 23 identical subcategories between sabinene and 4-terpineol, which indicates they had the closest relationship. This may be because they are both monoterpenes. In this study, our primary concern was the significantly enriched GO terms between the CSEO and the control. The significant changes in GO terms in biological processes mainly included multi-organism processes, interspecies interactions between organisms, and pathogenesis; cellular components mainly included the membrane part, intrinsic component of the membrane, cell periphery, and plasma membrane, and the molecular functions mainly included the activities of the transporters, oxidoreductase, and transmembrane transporter.

Furthermore, KEGG enrichment analysis was performed to investigate the metabolic pathways, and scatter plots of all significantly enriched KEGG pathways are shown in Figure 4D–F. The KEGG analysis for *C. albicans* treated with CSEO showed that all significantly enriched pathways of DEGs were mainly involved in metabolic pathways (82 genes), the biosynthesis of secondary metabolites (37 genes), and antibiotic biosynthesis (30 genes) (Figure 4F). There were fewer gene counts for the significantly enriched metabolic pathways of *C. albicans* treated with sabinene and 4-terpineol.

**Figure 5:**
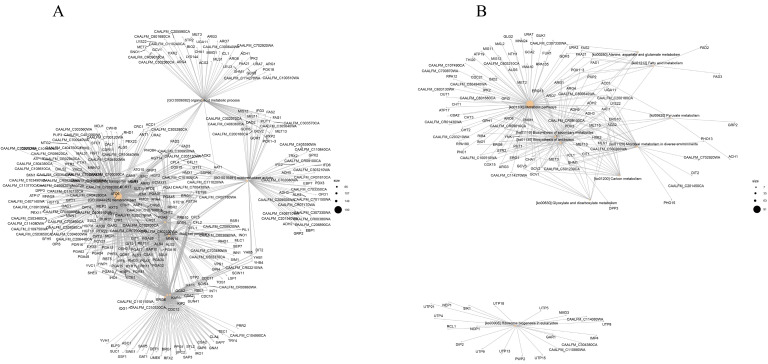
Network diagrams of the top 10 involved genes in the GO enrichment analysis of DEGs (**A**) and KEGG enrichment analysis of DEGs (**B**).

To reveal the genes related to CSEO, the top 10 DEGs of the GO and KEGG enrichment analyses between the CSEO and the control were selected to draw a network diagram showing the involved genes. As shown in Figure 5A, the functions of DEGs associated with the expression of *C. albicans* genes mainly focused on biofilm formation, such as the membrane, the intrinsic components of the membrane, the cell periphery, and the plasma membrane. Biofilm formation in *C. albicans* involves complex processes, including cell adhesion, hyphae growth, maturation, extracellular matrix secretion, and cell dispersion. Biofilm formation is also related to the development of drug resistance [18,23,24,25]. The most involved DEGs of the GO enrichment analysis were *AAP1*, *ACC1*, *ALS2*, *ALS3*, *ALS4*, *CSA1*, *CFL1*, *CFL2*, *CFL5*, *CRH11*, *ERG1*, *ECE1*, *FGR41*, *FET34*, *FET 99*, *GIT1*, *GIT2*, *HWP1*, *HYR1*, *IHD1*, *OPI3*, *OPT2*, *OPT4*, *PGA7*, *PGA11*, *PGA13*, *PGA15*, *PGA16*, *PGA17*, *PGA28*, *PGA32*, *PGA34*, *PGA37*, *PGA45*, *PGA54*, *RHD3*, *SAP10*, *SSU1*, and *YWP1*. Many of these genes have been reported in previous studies. For example, the agglutinin-like sequence (*ALS*) protein family is a type of cell wall glycoprotein, which is encoded by *ALS* genes, that regulates cell adhesion [26,27]. *ALS3*-encoded *Als3p* belongs to glycosyl phosphatidyl inositol (GPI) ankyrin, which is the most closely related to cell adhesion [23,28]. Loss of *ALS3* can lead to serious biofilm defects. *HWP1* is another important cell-adhesion-related gene [23,28,29]. The acidic mannoprotein *HWP1P* encoded by *HWP1* also belongs to GPI ankyrin, which is necessary for mycelial development and the adhesion of *C. albicans* to host cells. In addition, the family of secreted aspartyl proteases (*SAPs*) of *C. albicans* plays an important role in the pathogenic process connected to adhesion, immune escape, and tissue damage [30].

The network diagram of the top 10 DEGs of the KEGG enrichment analysis between CSEO and the control is shown in Figure 5B. The DEGs most involved in metabolic pathways, the biosynthesis of secondary metabolites, and antibiotic biosynthesis were *AAT1*, *ACC1*, *ACS2*, *ADH2*, *ADH3*, *ADH5*, *ARG1*, *ARG3*, *ARG4*, *ARO7*, *ARO8*, *CHA1*, *CIT1*, *COX15*, *ERG1*, *ERG10*, *ERG13*, *ERG6*, *GCV1*, *GCV2*, *GCV3*, *MLS1*, *OPI3*, *SHM1*, *STR2*, and *YNK1*. Among these, the alcohol dehydrogenase (*ADH*) gene family is soluble in hyphae and can induce Spider biofilm. *ADH1* and *ADH2* were detected during the transition of yeast into hyphae. Glutathionylation may be involved in the process of glycolysis metabolism [31]. An intracellular balance is required in hyphae formation, including minimal glutathione limitation. Like the *ADH* gene family, the *ARG* gene family is also Spider-biofilm-induced, which shows that CSEO may impact the metabolic pathways of biofilm formation. The GO and KEGG enrichment analyses also exhibited some of the same genes, such as *ACC1*, *ACS2*, *ERG1*, and *OPI3*, which may indicate that the functions and metabolic pathways were associated. Besides these previously reported genes, many other genes have not been reported, and further experimental confirmation is needed to study their specific mechanisms. These obtained genes must be verified using real-time quantitative PCR in the following study. Although quantitative and transcriptome analysis illustrated the importance of 4-terpineol in the anti-*C. albicans* activity of CSEO, other chemical components of CSEO may have anti-*C. albicans* activity or there may be synergism with other components since CSEO is a complex mixture. A further verification study will be conducted.

## 3. Materials and Methods

### 3.1. Chemicals and Plant Materials

The chemicals and their suppliers are listed as follows: sabinene (>75%), *α*-phellandrene (>85%), and 4-terpineol (>98%) (Sigma-Aldrich, Shanghai, China); *β*-phellandrene (>96%) (Toronto Research Chemicals TRC, Toronto, ON, Canada); *β*-caryophyllene (>90%) (TCI Chemical Industry Development Co., Ltd., Shanghai, China); fluconazole (Aladdin, Shanghai, China); dimethyl sulfoxide (DMSO) (Thermo Scientific Co., Ltd., Shanghai, China); *n*-hexane and anhydrous sodium sulfate (chromatographic purity, Aladdin, Shanghai, China); TRIzol Reagent (Magen); trichloromethane and isopropanol (Sinopharm); RNA Nano 6000 Assay Kit (Agilent Technologies, Santa Clara, CA, USA); NEBNext^®^ Ultra^TM^ RNA Library Prep Kit for Illumina^®^ (ABclonal, Boston, MA, USA); AMPure XP system (Beckman Coulter, Brea, CA, USA). Ultra-pure water was used throughout the experiment employing a RODI-160B1 ultra-pure water meter (Research Scientific Instruments Co., Ltd., Xiamen, China).

The fresh fruits of *Clausena lansium* (Lour.) Skeels were picked from Haikou, Hainan, China, in June 2021 and identified by Professor Weili Yang (Hainan Medical University) and stored in the Public Research Center of Hainan Medical University (Haikou, China).

### 3.2. Strains and Culture Conditions

The standard reference strain *Candida albicans* (Robin) Berkhout (*C. albicans* SC5314) was obtained from Beijing Baiou Bowei Biotechnology Co., Ltd. (Beijing, China). The clinical strain (*C. albicans* 27) was obtained from patients with oral candidiasis at Wenchang General Hospital (Wenchang, China). The identification of the clinical isolate was based on ITS gene sequence analysis. These strains were stored in a −80 °C freezer before use. They were activated in Sabouraud dextrose agar (SDA) (Huankai Microbial Sci. and Tech. Co., Ltd., Guangzhou, China) at 35 °C for 24 h. RPMI 1640 medium (Thermo Scientific Co., Ltd., Shanghai, China) was used in the broth dilution method and the kinetic growth curves.

### 3.3. Extraction of CSEO

The *C. lansium* seeds were extracted from the fruits and washed. After drying in an SPX-250B-Z biochemical incubator (Bo Xun Industrial Co., Ltd., Shanghai, China) at 40–50 °C, the seeds were crushed using an FW80 high-speed grinder (Taisite Instrument Co., Ltd., Tianjin, China) and soaked in water with a material-to-solvent ratio of 1:8 (*w*/*v*) for 5.0 h. Hydrodistillation was used to extract the CSEO using a Clevenger-type apparatus for 2.5–3.0 h. The yield of CSEO was calculated according to the ratio of the extracted volume reading from the scale of the apparatus to weight. The obtained CSEO was dried using anhydrous sodium sulfate and stored at 4 °C in a freezer before being analyzed.

### 3.4. GC–MS Analysis of the Five Components of CSEO

The samples were analyzed using a GCMS-QP2010 Plus (Shimadzu, Kyoto, Japan) with a DB-5MS column (30 m × 2.5 mm; 0.25 μm film thickness; Agilent, USA). Helium was used as the carrier gas at a flow rate of 1.0 mL·min^−1^ in split mode (1:20). A temperature program was used, starting at 60 °C and increasing to 100 °C at a rate of 5 °C·min^−1^, then increasing to 220 °C at a rate of 20 °C·min^−1^, and finally remaining at this temperature for 2 min. The temperatures of the injector, the connector, and the source were 250 °C, 250 °C, and 280 °C, respectively. MS with impact ionization (EI) mode was used at 70 eV. First, the retention time and the fragment ions were obtained in full-scan mode (*m*/*z* 50–500) by injecting the standard solution. Then, according to the full-scan results, one quantitation ion peak and two qualification ion peaks were monitored for each component. The quantitation peak (*m*/*z*) of sabinene, *α*-phellandrene, and *β*-phellandrene was 136, and the qualifying icons (*m*/*z*) were 93 and 77. The quantitation icon (*m*/*z*) of 4-terpineol and *β*-caryophyllene were 154 and 204, respectively. The qualifying icons (*m*/*z*) of 4-terpineol were 93 and 71, and those of *β*-caryophyllene were 93 and 69.

### 3.5. Determination of Minimal Inhibitory Concentrations (MICs) and Minimum Fungicidal Concentrations (MFCs)

The MICs of CSEO, sabinene, and 4-terpineol against *C. albicans* SC5314 and *C. albicans* 27 were determined using the broth dilution method according to the CLSI M27-A3 guidelines [32]. The 96-microwell plates were prepared at different concentrations with CSEO, sabinene, and 4-terpineol solutions (with <1% DMSO). The negative control (without inoculation) and the positive control (with fluconazole) were needed. The concentration of the yeast cells was adjusted to 1–5 × 10^3^ CFU·mL^−1^ with RPMI 1640 medium. The OD values were measured at 600 nm using a BioTek Synergy HTX enzyme-linked immunosorbent assay (ELISA, America Berten Instrument Co., Ltd., Burlington, VT, USA) after incubation at 35 °C for 48 h. The MIC was determined as the minimum concentration that caused 50% inhibition. Finally, after 72 h of cultivation, 20 μL solutions from each negative control and test well were poured onto an SDA plate. The minimum concentration when the colony number was no more than 3 CFU following incubation at 35 °C for 48 h was the MFC.

### 3.6. Effects of CSEO, Sabinene, and 4-Terpineol on the Kinetic Growth of C. albicans Strains

The effects of CSEO, sabinene, and 4-terpineol on the kinetic growth of *C. albicans* SC5314 and *C. albicans* 27 were tested. After being incubated to the logarithmic stage for 16 to 20 h at 35 °C, 100-microwell plates were prepared with the three tested solutions at different concentrations (1/2 MIC, MIC, and MFC) and incubated in a Bioscreen C automatic growth curve analyzer (Bioscreen, Helsinki, Finland) at 35 °C and 200 rpm. The kinetic growth of the *C. albicans* strains was evaluated by measuring the OD values at 600 nm every 2 h of incubation. All values were measured in triplicate.

### 3.7. Transcriptome Determination

The total RNA was extracted from the *C. albicans* SC5314 cells exposed to the MICs of CSEO, sabinene, and 4-terpineol for 1 h using TRIzol Reagent following the manufacturer’s instructions. *C. albicans* SC5314 cells without treatment were used as the control. The RNA samples were qualified with the integrity and total amount of RNA as the main reference indicators using a NanoDrop ND-2000 system (Thermo Scientific, Waltham, MA, USA) and an Agilent Bioanalyzer 4150 system (Agilent Technologies, Santa Clara, CA, USA). Paired-end libraries were prepared using the TruSeq RNA Sample Preparation Kit according to the manufacturer’s instructions. Finally, the library preparations were sequenced using an Illumina 6000 (Illumina, San Diego, CA, USA).

### 3.8. Bioinformatics Transcriptome Analysis

The data generated from the Illumina platform were used for bioinformatics analysis. All of the analyses were performed using an in-house pipeline from Shanghai Applied Protein Technology. Then, mapped reads were obtained by aligning clean reads with the reference genome in orientation mode separately using the HISAT2 software (http://daehwankimlab.github.io/hisat2/, accessed on 24 July 2020). The read numbers mapped to each gene were counted using Feature Counts (http://subread.sourceforge.net/, accessed on 15 July 2021). The fragments per kilobase of exon model per million mapped reads (FPKMs) were calculated using the length of each gene and the count of mapped reads. Differential expression analysis was performed using DESeq2 (http://bioconductor.org/packages/release/bioc/html/DESeq2.html, DESeq2 package version: 1.42.0). Significantly different expression was defined as both differential expression genes (DEGs) having a |log2 (foldchange)| of >1 and Padj of <0.05. To further understand the functions and enrichment pathways involved for these DEGs, Gene Ontology (GO) and Kyoto Encyclopedia of Genes and Genomes (KEGG) analyses were performed.

### 3.9. Statistical Analysis

Each experiment was carried out with three biological and technical replicates. The data are presented as mean values ± standard deviation. The significant difference between groups was determined using a two-way analysis of variance (two-way ANOVA). A *p* of <0.05 was considered to achieve significant enrichment. GraphPad Prism 6 (GraphPad Software Inc., San Diego, CA, USA) was used for drawing the graphs.

## 4. Conclusions

This is the first study on the investigation of the antifungal mechanisms of CSEO against *C. albicans* based on quantitative analysis using GC–MS and transcriptomics analysis using RNA-seq. Five volatile components including sabinene, *α*-phellandrene, *β*-phellandrene, 4-terpineol, and *β*-caryophyllene in CSEO were analyzed with the standard curve method using GC–MS. The MICs and MFCs of CSEO, sabinene, and 4-terpineol were in the ranges of 0.25–2.00 mg·mL^−1^ and 0.50–4.00 mg·mL^−1^, respectively. The effects of CSEO, sabinene, and 4-terpineol on the kinetic growth of *C. albicans* further proved that the antifungal activity of CSEO was better than that of sabinene and 4-terpineol against fluconazole-resistant *C. albicans*. Additionally, the Venn diagram and clustering analysis of the DEGs of *C. albicans* cells exposed to CSEO, sabinene, and 4-terpineol indicated that the anti-*C. albicans* mechanism of CSEO might be similar to that of 4-terpineol from the perspective of genes using RNA-seq. Via GO and KEGG enrichment analyses, the DEGs in *C. albicans* treated with CSEO were mainly concentrated in membrane-related cellular components and metabolic pathways, which may be one of the important reasons for CSEO inhibiting the growth of fluconazole-resistant *C. albicans*. In conclusion, quantitative and transcriptomics analysis illustrated the importance of 4-terpineol to anti-*C. albicans* activity in CSEO. The obtained results of this study may promote a better understanding of the anti-*C. albicans* mechanism of CSEO and provide direction for further research and the development of *C. lansium* seeds for treating *C. albicans* infection, especially the azole resistance of *C. albicans* infection.

## Figures and Tables

**Table 1:** Retention times, linear ranges, slopes, intercepts, *r* values, LOQs, and LODs for the five components using GC–MS.

Component	Retention Time (min)	Linear Range (μg·mL^−1^)	Slope	Intercept	*r*	LOQ (ng·mL^−1^)	LOD (ng·mL^−1^)
Sabinene	5.89	0.25–31.50	15,960	198.14	0.9998	50.40	10.08
*α*-phellandrene	6.65	0.28–35.02	13,466	−1049.30	0.9990	56.03	11.21
*β*-phellandrene	7.25	0.27–33.55	10,534	565.17	0.9997	53.68	10.74
4-terpineol	10.15	0.26–32.93	8789.5	−660.18	0.9999	52.68	10.54
*β*-caryophyllene	12.65	0.25–32.40	5958.1	1349.6	0.9997	103.68	20.74

## Data Availability

Data are contained within the article and supplementary materials.

## Supplementary Materials

The following supporting information can be downloaded at https://www.mdpi.com/article/10.3390/molecules28248052/s1, Table S1: Summary statistics of RNA-seq transcriptome in this study.

## Abbreviations

EO	Essential oil
CSEO	Essential oil of *Clausena lansium* (Lour.) Skeels seeds
GC-MS	Gas chromatography–mass spectrometry
DMSO	Dimethyl sulfoxide
SDA	Sabouraud dextrose agar
EI	Electron impact ionization
MIC	Minimal inhibitory concentration
MFC	Minimum fungicidal concentration
FPKM	Fragments per kilobase of exon model per million mapped reads
DEGs	Differential expression genes
GO	Gene Ontology
KEGG	Kyoto Encyclopedia of Genes and Genomes
LOQ	Limit of quantification
LOD	Limit of detection
S/N	Signal-to-noise ratio
*r*	Correlation coefficient
RSD	Relative standard deviation value
BP	Biological process
CC	Cellular component
MF	Molecular function
